# ANGPTL3 Overexpression Suppresses the Development of Oncogenic Properties in Renal Cell Carcinoma via the Wnt/*β*-Catenin Signaling Pathway and Predicts Good Prognosis

**DOI:** 10.1155/2021/2863856

**Published:** 2021-08-24

**Authors:** Yu-jian Zhang, Lin Zhang, Fei Feng, Qi-feng Cao

**Affiliations:** ^1^Department of Urology, Dafeng People's Hospital, Dafeng, 224100 Yancheng, Jiangsu, China; ^2^Department of Urology, The Xin Hua Hospital Affiliated to Shanghai Jiao Tong University School of Medicine, 200092, Yangpu, Shanghai, China

## Abstract

Angiopoietin-like 3 (ANGPTL3), which is involved in new blood vessel growth, has been reported to exhibit an abnroaml expression in many different cancers. However, the expressing pattern and functions of ANGPTL3 renal cell carcinoma (RCC) were rarely reported. In this study, we observed that ANGPTL3 expression was distinctly downregulated in both RCC specimens from TCGA datasets and cell lines. Survival assays also revealed that patients with low ANGPTL3 expression exhibited a shorter overall survival and disease-free survival than those with high ANGPTL3 expression. Cell counting kit-8 (CCK-8) assay, Colony formation assay, and flow cytometry showed that overexpression of ANGPTL3 distinctly suppressed the proliferation of RCC cells, and promoted apoptosis. Transwell assays and Wound healing assays revealed that ANGPTL3 upregulation suppressed the migration and invasion of RCC cells. Then, we explored whether ANGPTL3 dysregulation influenced the alteration of Wnt/*β*-catenin signaling using TOP/FOP flash reporter assays and western blot. The results showed that overexpression of ANGPTL3 distinctly suppressed the activity of Wnt/*β*-catenin signaling. Overall, our results confirmed that overexpression of ANGPTL3 was related to the malignancy and good prognosis of RCC patients, and ANGPTL3 upregulation inhibited the tumor proliferation and metastasis via the Wnt/*β*-catenin pathway. ANGPTL3 may be a novel therapeutic target and a prognostic biomarker for RCC patients.

## 1. Introduction

Renal cell carcinoma (RCC), accounting for >4% of adult neoplasms, is the most common malignant tumor of the kidney in adults, with a mortality rate (approximately 45%) [1, 2]. The largest subtype of RCC is clear cell RCC (>70%) [3]. RCC includes several histological subtypes possessing obvious biological characteristics and clinical outcomes [4]. Early detection displays a significant benefit for the long-term survival of RCC patients, and patients diagnosed with organ-confined diseases show a five-year survival of >85% [5–7]. However, for those patients with positive metastasis, the 5-year survival is only approximately 10% [8]. Therefore, there is a crucial need to found new biomarkers and targeted therapies for this aggressive malignancy.

Angiopoietin-like protein 3 (ANGPTL3), located on 1p31.3 and also known as angiopoietin-5, is intimately associated with the disorders of lipid metabolisms [9]. ANGPTL3 are functionally defined by the C-terminal fibrinogen-like domain which has been confirmed to exhibit regulatory functions via the modulation of the Tie2 receptor [10]. The above function allows ANGPTL3 to enhance several fundamental events involved in angiogenesis [11]. In recent years, increasing researches suggested the critical roles of ANGPTL3 in the regulation of various vital movements, such as haematopoietic functions, angiogenesis and lipid metabolisms [12, 13]. In addition, several pathological changes, such as liver diseases, diabetes, carcinogenesis and atherosclerosis, are also reported to be regulated by the dysregulation of ANGPTL3 [14–16]. In recent years, more and more studies have confirmed that ANGPTL3 expression was dysregulated in several types of tumors and exhibited regulatory effects on the development and progression of tumors [17–19]. These findings suggest ANGPTL3 as a novel biomarker and therapeutic target for tumor patients.

Previously, ANGPTL3 was found to be lowly expressed in RCC and suppress the metastasis of RCC cells via inhibiting VASP phosphorylation [20]. In addition, it was proved that ANGPTL3 could regulate the sensitivity of sorafenib in RCC by suppressing p53 ubiquitination mediated by FAK [21]. However, the expressing pattern, function, and the potential mechanisms of ANGPTL3 in RCC were rarely reported. In this study, furtherer evidences that the expression of ANGPTL3 was decreased in RCC were provided. Then, we further explored the tumor-related functions and molecular mechanisms of ANGPTL3 in RCC progression.

## 2. Materials and Methods

### 2.1. Cell Cultures Transfection

The human renal tubular epithelial cell line (HK-2) and human RCC cell lines (786-O, Caki-1, A498) were both purchased from the Institute of Cell Research(Shanghai, China). DMEM contained with 10% (v/v) FBS, 100 U/ml penicillin, and 100 *μ*g/ml streptomycin were used to maintain the cells, which cultured in a moist 5% CO_2_ atmosphere at 37°C. Cells were subcultured at 80% to 90% confluency. To ectopic the expressions of ANGPTL3 in RCC cells, the expressing plasmid for ANGPTL3 were PCR-amplified and subcloned into the pcDNA3.1 vector (PPL50117-2a, Yipu, Wuhan, Hubei, China). An empty pcDNA 3.1 vector was used as a control. Transfection of ANGPTL3 as conducted using the Lipofectamine RNAiMAX transfection reagent (Thermo fisher Scientific, Waltham, MA, USA) according to the manufacturer's protocol.

### 2.2. Extraction of Total RNA and qRT-PCR

Total RNAs were extracted with 1 mL TRIzol (Invitrogen), and the total RNAs were reversed to cDNA by PrimeScript RT kit (Takara, Zhejiang, Hangzhou, China). Based on the product guide, Prime Script-RT reagent Kit and SYBR Premix ExTaq (Takara, Zhejiang, China) were applied to perform PCR assays, for the purpose of detecting ANGPTL3 expression. The primers were as shown: ANGPTL3 sense: 5'- ATTTTAGCCAATGGCCTCCTTC-3'; ANGPTL3 antisense: 5'- CTGGTTTGCAGCGATAGATCATA-3'; GAPDH sense: 5'-AGAAGGCTGGGGCTCATTAC-3'; GAPDH antisense: 5'-AGGGGCCATCCACAGTCTCCA-3'. Data were assessed using the 2^-*ΔΔ*CT^ methods.

### 2.3. CCK-8 Assays

To evaluate cell proliferation, Cell Counting Kit-8 (CCK-8) (Dojindo, Japan) was used. The overexpression transfected 786-O and A498 cells, seeded on 96-well plates, were cultured for 0 h, 24 h, 48 h, 72 h, 96 h, separately. At special points in time, a total of 10 *μ*l of CCK-8 regent (Sigma, Shenzhen, Guangdong, China) was added in corresponding wells, followed by incubation for 6 hours. Under 450 nm wavelength, a micro-plate reader was applied to examine the OD.

### 2.4. Colony Formation Assay

A 3.5 cm cell culture dish (Corning, Chengdu, Sichuan, China) was used to seeding the cells. The 200 *μ*L DMEM containing 10% FBS, working as the medium that volatilized into the incubator, was supplemented into the dish every 2 days. After 2 weeks, the colonies were visible to the naked eye. Next, 95% methanol was used to fix the cells which were further stained by the use of methyl violet(C0089, Baomanbio, Xuhui, Shanghai, China). Under an IX71 inverted microscope, our group counted the tumor colonies (>50 cells).

### 2.5. Apoptosis Assays

Cell apoptosis was detected via FACScan flow cytometer(BD Biosciences, China). Propidium iodide (PI) and Annexin V (BestBio, China) were used for the stain of the collected cells.

### 2.6. Transwell Assay

24-well plate Boyden chamber with a hole membrane of 8 *μ* m was used for the invasion assay detection of 786-O and A498. 40 *μ*l Matrigel was used to coat the membranes. 1 × 10^4^ cells were placed in the upper chamber of each Transwell. 0.5 *μ* g/mL medium was added to the lower Boyden cavity. Twenty-four hours later, we wiped out the non-invaded cells. Subsequently, we fixed the filters, followed by stain by the application of crystal violet staining. An inverted microscope was applied for the calculation of the cells.

### 2.7. Wound Healing Assay

Six-well plates were used to incubate cells. When the cellular adherence reached 85%, a 10 *μ*l sterile pipette tip was used to scratch the cellular layer. After the old medium was abandoned, PBS was used to wash the shed cells. The time of wound infliction was considered as 0 h, and a microscope with a camera was applied for photograph of wound closure. ImageJ 1.50v was used to quantify the areas covered by migrated cells. All the experiments were repeated in 3 times.

### 2.8. TOPFlash Luciferase Assays

Cells were planted in 24-well plates. Wnt/*β*-catenin TOPFlash plasmids (Yiqiao Biology, Yizhuang, Beijing, China) and mutant FOPFlash plasmids (Addgene, Cambridge, MA, U.S.A.) and Renilla TK-luciferase vector (Promega, Haidian, Beijing, China) were transfected into the cells together. Subsequently, luciferase detection kits (Promega, Haidian, Beijing, China) were applied to assess the cellular luciferase activity.

### 2.9. Western Blot Assays

By the use of RIPA buffer, we collected the total proteins of cells, and BCA protein detection kit was applied for the examination of the protein-related concentration. The same amounts of proteins were separated by SDS/PAGE and transferred to 0.22 *μ*m PVDF membranes. Before incubated with *β*-catenin, cyclin D1 and C-myc at 4°C overnight, these membranes were blocked. Then, the membrane was incubated with HRP-conjugated anti-mouse or -rabbit secondary antibody for 1 h at room temperature. ECL kit helped to observe the protein band.

### 2.10. Statistical Analysis

GraphPad Prism 5 was used for statistical analyses. Data were expressed as mean ± SD. Student's t test was used to compare the differences between the groups. P <0.05 was statistically significant.

## 3. Results

### 3.1. High Levels of ANGPTL3 in RCC and Its Prognostic Value

**Figure 1:**
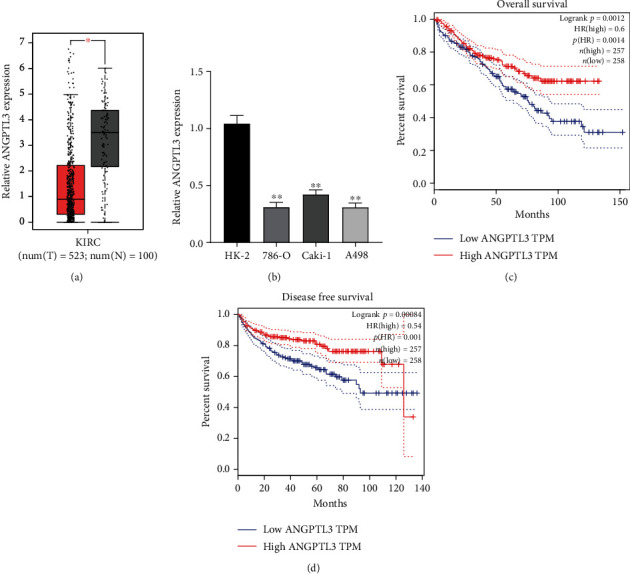
ANGPTL3 was lowly expressed in RCC. (a) Higher levels of ANGPTL3 were observed in RCC specimens from TCGA datasets. (b) RT-PCR determined the expression of three RCC cells and HK-2 cells. (c) Overall survival of 315 RCC patients based on GEPIA results. (d) Disease-free survival of 315 RCC patients based on GEPIA results. ∗∗p <0.01, ∗p <0.05. The experiments were repeated thrice, in triplicates.

To screen the possible functional regulator involved in RCC progression, we searched “GEPIA” (A online tool analyzing TCGA datasets) [22], finding that the expression of ANGPTL3 was distinctly decreased in RCC specimens compared with non-tumor renal specimens (Figure 1(a)). Then, we performed RT-PCR using RCC cell lines and found that ANGPTL3 levels were distinctly decreased in RCC cells compared with HK-2 cells (Figure 1(b)). The prognostic value of ANGPTL3 in RCC was explored by analyzing survival data from TGCA datasets(515 RCC patients). The result showed patients with higher ANGPTL3 expressions had a shorter overall survival(p =0.0014, Figure 1(c)) and disease-free survival(p =0.00084, Figure 1(d)) than those with lower ANGPTL3 expressions. Our findings suggested ANGPTL3 as a prognosis-related regulator in RCC.

### 3.2. Overexpression of ANGPTL3 Suppressed the Proliferation and Metastasis of RCC Cells

**Figure 2:**
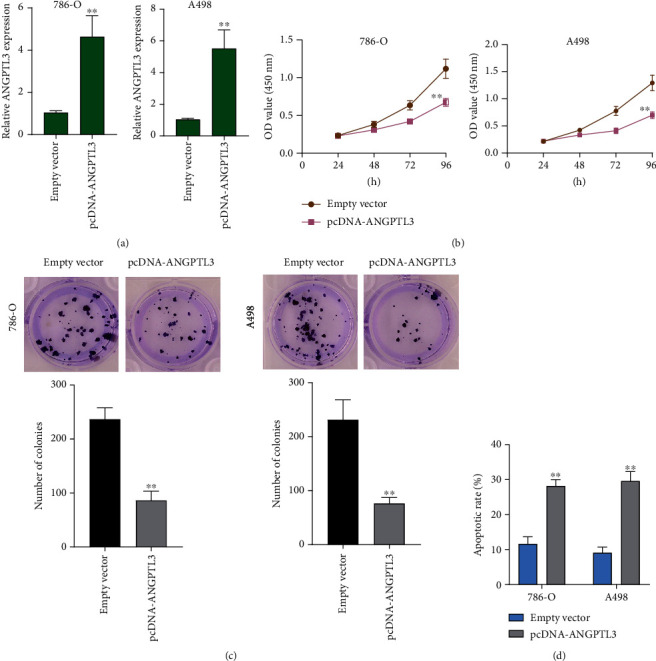
Overexpression of ANGPTL3 suppressed proliferation of RCC cells. (a) A498 and 786-O cells were transfected with pcDNA-ANGPTL3 or empty vector, and qRT-PCR was conducted to confirm the transfection efficiency. (b) An CCK-8 assay showed that ANGPTL3 expression reduced cell proliferation. (c) Using Cell colony formation assays, cellular numbers were calculated after ANGPTL3 overexpression. (d) The apoptosis rate of ANGPTL3-overexpressed RCC cells distinctly increased. ∗∗p <0.01. The experiments were repeated thrice, in triplicates.

**Figure 3:**
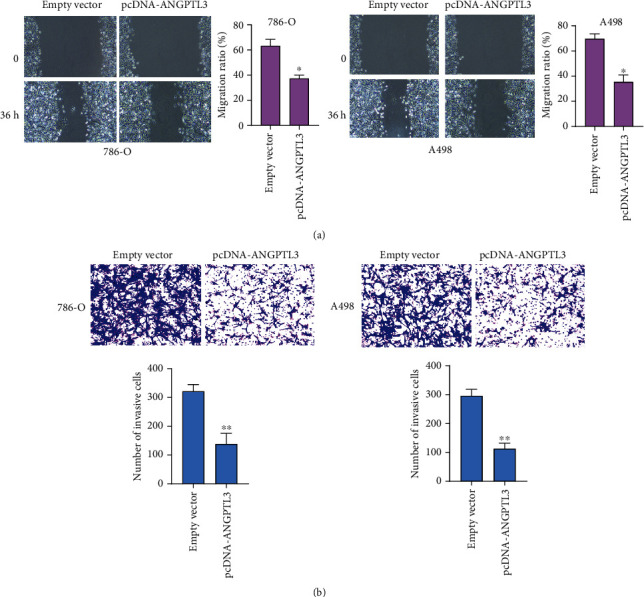
Overexpression of ANGPTL3 inhibited the metastasis of A498 and 786-O cells. (a) Wound healing assays were conducted to evaluate cell mobility after ANGPTL3 overexpression. (b) Transwell assays were performed to assess cell invasion after ANGPTL3 overexpression. ∗∗p <0.01. The experiments were repeated thrice, in triplicates.

Then, gain-of-function assays were conducted to find out whether ANGPTL3 upregulation influenced the RCC ability. By the use of pcDNA-ANGPTL3, ANGPTL3 was overexpressed in A498 and 786-O cells, which was proved by RT-PCR (Figure 2(a)). CCK-8 assays results suggested that the OD value of A498 and 786-O cells transfected with pcDNA-ANGPTL3 at 450 nm was distinctly lower than that of cells transfected with empty vector (Figure 2(b)). Colony formation assays also confirmed that ANGPTL3 overexpression distinctly increased number of colonies (Figure 2(c)). The results of flow cytometry showed that compared with empty vector-transfected RCC cells, ANGPTL3-overexpressed 786-O and A498 cells showed increased apoptotic rates (Figure 2(d)). In addition, to explore the impacts of ANGPTL3 on the metastasis ability of RCC cells, Wound healing assays and Transwell assays were carried out. As shown in Figure 3(a), we observed that the migrative ratio of RCC cells transfected with pcDNA-ANGPTL3 was distinctly reduced than those transfected with empty vector. Moreover, overexpression of ANGPTL3 was also observed to reduce the number of invasive cells (Figure 3(b)). Overall, our findings suggested that ANGPTL3 served as a tumor promotor in RCC.

### 3.3. Overexpression of ANGPTL3 Suppressed the Activity of Wnt/*β*-Catenin Pathway

**Figure 4:**
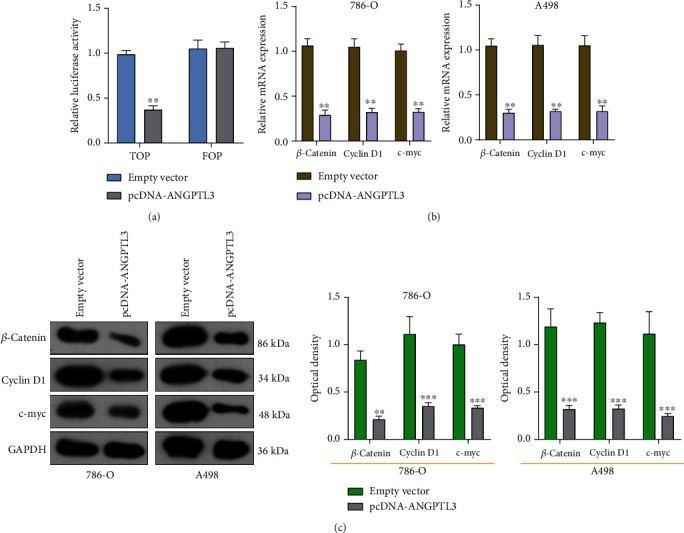
Overexpression of ANGPTL3 affected the Wnt/*β*-catenin pathway. (a) *β*-catenin TCF/LEF promoter activity in 786-O cells was determined by TOP-Flash luciferase reporter assays. (b) The expressions of c-myc, cyclin D1 as well as *β*-catenin were examined in A498 and 786-O cells after ANGPTL3 overexpression. (c) Western blot for the levels of c-myc, cyclin D1 as well as *β*-catenin in RCC cells after ANGPTL3 overexpression. ∗∗p <0.01. The experiments were repeated thrice, in triplicates.

In order to study the mechanisms of ANGPTL3 in the progression of RCC, we deeply studied the dysregulation of Wnt/*β*-catenin pathway in RCC cells. We first carried out TOP/FOP flash reporter assays using 786-O cells. Our data revealed that the luciferase activity of RCC cells with pcDNA-ANGPTL3 overexpression was distinctly decreased compared with those transfected with empty vector (Figure 4(a)). Then, we performed RT-PCR to study the influences of ANGPTL3 overexpression on Wnt/*β*-catenin pathway and observed that ANGPTL3 overexpression distinctly suppressed the expressions of Wnt-related proteins(Cycline D1, c-myc, B-Caternin in A498 and 786-O cells) (Figure 4(b)), which was also demonstrated by the use of western blot assays (Figure 4(c)). Our findings suggested ANGPTL3 may exhibit its oncogenic roles in RCC via modulating Wnt/*β*-catenin pathway.

## 4. Discussion

RCC incidence has increased for over two decades [23]. Current therapeutic tools are effective in patients diagnosed at early stages, but there are limited treatment options for patients with advanced stages [24, 25]. In order to improve prognosis of RCC patients, the investigation about the prognostic factors for RCC is especially important, because such predictors are helpful in guiding clinical management [26, 27]. In this study, we found that the levels of ANGPTL3 were down-regulated in the TGCA dataset specimens and cell lines. Previously, the distinct downregulation of ANGPTL3 was also observed in ovarian carcinoma. However, its upregulation was observed in oral cancer, esophageal cancer and hepatocellular carcinoma, suggesting its variety in tumor development [18, 19, 28]. We analyzed TCGA datasets to research the prognostic value of ANGPTL3 for RCC patients and found that patients with higher ANGPTL3 expression exhibited a shorter OS and DFS of RCC patients. For RCC patients, ANGPTL3 may be a new prognostic biomarker. However, more clinical samples with survival assays are needed to deeply explore the prognostic value of ANGPTL3 in RCC patients.

In the past years, many studies have revealed the functions of ANGPTL3 in tumor progression. For instance, ANGPTL3 was found to display a high level in oral cancer and possess a potential diagnostic value according to the results of ROC assays. Functionally, knockdown of ANGPTL3 was shown to suppress the proliferation of oral cancer cells via activating ERK/MAPK pathway [18]. Yu and his group reported that ANGPTL3 was overexpressed in hepatocellular carcinoma. They observed that downregulation of ANGPTL3 inhibited cell proliferation and decreased invasion of hepatocellular carcinoma cells [29]. These findings suggested ANGPTL3 as an oncogene in the above tumors. However, a previous study by Zhao et al. reported that overexpression of ANGPTL3 resulted in the distinct enervation of metastasis of RCC cells [20]. In this study, we also discovered that ANGPTL3 overexpression inhibited the activities of RCC cells, such as proliferation, migration, and invasion. These findings were consistent with previous findings.

The Wnt signaling pathway is a key regulatory pathway for a variety of biological progresses including embryonic development, differentiation, proliferation, and adult tissue maintenance [30]. Excessive activation of Wnt signaling has been found in many kinds of tumors, which gives cells the ability to increase tumorigenicity, continue to proliferate, and enhance the potential for metastasis [31, 32]. The Wnt/*β*-catenin signaling pathway is very important in RCC pathogenesis [33]. In this study, we used TOP/FOP flash reporter assays which confirmed that overexpression of ANGPTL3 inhibited the activation of the Wnt/*β*-catenin signaling. Then, we examined the effect of ANGPTL3 dysregulation on the Wnt/*β* catenin signaling pathway, finding that overexpression of ANGPTL3 suppressed c-myc, cyclin D1 and *β*-catenin. Our findings suggested ANGPTL3 may exhibit its oncogenic roles via modulating Wnt/*β* catenin signaling.

## 5. Conclusion

In summary, ANGPTL3 was lowly expressed in RCC and predicted a poor prognosis for RCC patients. Overexpression of ANGPTL3 suppressed RCC progression through inhibiting Wnt/*β* catenin signaling. This study revealed the vital significance of ANGPTL3 in RCC development. However, more specimens were needed to further confirm our findings, and the potential mechanisms involved in ANGPTL3 function were needed to be further studied.

## Data Availability

The data used to support the findings of this study are available from the corresponding authors upon request.
